# The Digital Assessment of Cognition: Informant Ratings of Functional Disabilities and Psychiatric Symptoms

**DOI:** 10.1093/geroni/igaf122.2163

**Published:** 2025-12-31

**Authors:** David Libon, Rod Swenson, Sheina Emrani, Terri Ginsberg, Mitchel Kling, Kevin Overbeck, Lenny Powell, Adaora Okoli-Umeweni

**Affiliations:** Rowan University, Glassboro, New Jersey, United States; North Dakota Medical School, Grand Forks, North Dakota, United States; University of Pennsylvannia, Philadelphia, Pennsylvania, United States; Rowan University, Glassboro, New Jersey, United States; Rowan-Virtua School of Osteopathic Medicine, Stratford, New Jersey, United States; Rowan-Virtua School of Osteopathic Medicine, Stratford, New Jersey, United States; Rowan-Virtua School of Osteopathic Medicine, Stratford, New Jersey, United States; Rowan University, Glassboro, New Jersey, United States

## Abstract

**Background:**

The Digital Cognitive Assessment (DAC) is a 7-minute, iPad administered/scored protocol that assesses verbal episodic memory, verbal working memory, and language-related skills. The current research examined relations between DAC-memory and executive indices and family ratings for neurocognitive decline, instrumental activities of daily living (IADL) impairment, and psychiatric symptoms.

**Methods:**

179 memory clinic patients were assessed with the DAC. Spouses or knowledgeable family members rated the severity of neurocognitive impairment, IADL decline, and psychiatric symptoms using the Everyday Cognition Scales (Ecog); the Functional Assessment Questionnaire (FAQ) and the Instrumental Activities of Daily Living – Compensation Scale (IADL-C); and the Neuropsychiatric Inventory (NPI).

**Results:**

Partial correlations controlled for age, education, and sex found that greater informant total Ecog, FAQ and IADL-C scores were associated with lower DAC Memory and Executive summary scores. Greater IADL-C Money/Self-Management difficulty was associated with a lower DAC Memory summary score; while greater IADL-C Social Skills difficulty was associated with a lower DAC Executive summary score. A lower DAC Executive summary score was associated with greater informant rated NPI-apathy.

**Conclusions:**

The Digital Assessment of Cognition is powerful tool to assess neurocognitive abilities. The relations between DAC summary scores, total informant FAQ and IADL- scores; and IADL-C subscales and selected NPI-defined psychiatric problems suggest that DAC assessment of memory and executive abilities are both sensitive to gross IADL decline, and specific to differing IADL-C difficulty and selected psychiatric problems. Combining the DAC with informant IADL ratings is an effective strategy to assess for emergent MCI and dementia syndromes.

